# What kind of information do early parental report instruments provide on language ability at 3;6 when used at 2;0? A longitudinal comparison study

**DOI:** 10.3389/fpsyg.2023.1206949

**Published:** 2023-07-20

**Authors:** Susanna Surakka, Suvi-Maria Vehkavuori, Katri Saaristo-Helin, Suvi Stolt

**Affiliations:** Department of Psychology and Logopedics, University of Helsinki, Helsinki, Finland

**Keywords:** parental report instrument, language development, receptive language, early screening, language assessment, communicative development inventories, CSBS infant-toddler checklist, longitudinal study

## Abstract

**Introduction:**

Various parental report instruments are available for assessing children’s language skills at the end of the second year. However, comparison studies on their usability are lacking, and it is also open to question what kind of information the instruments provide when used in a parallel manner. This longitudinal study investigated which of the available three parental report instruments, when used at 2;0 (year;month), provides the most representative information on language development at 3;6. In addition, since most of the parental report instruments available focus specifically on expressive language, the role of receptive language ability was also investigated when analyzing the explanatory value of parental report instruments.

**Methods:**

The participants were 68 typically developing children. At 2;0, language skills were measured using the following measures: the Infant-Toddler Checklist of the Communication and Symbolic Behavior Scales Developmental Profile (ITC), the Short Form and Long Form versions of the Finnish Communicative Development Inventories (FinCDI-SF, FinCDI-LF), and the Reynell Developmental Language Scales III (RDLS). The outcome measures were receptive/expressive/ general language ability at 3;6 measured using RDLS.

**Results:**

The results of parental report instruments were significantly and positively associated with language ability at 3;6. The correlation between the combined value of ITC and FinCDI-SF and later language ability was stronger than correlations for each measure separately. The regression models with the results of parental report instruments as predictors explained 18–22% (p < 0.00) of the variability in the total RDLS score. However, when receptive language ability at 2;0 was included in the models as a predictor, R2 increased considerably (46–48%, p < 0.00).

**Discussion:**

The results adduce the usability of parental report measures along with the importance of measuring receptive language skills at 2 years of age. In summary, this study provides important insights into the clinical evaluation of early language ability.

## 1. Introduction

Parental report instruments are useful for investigating young children’s language abilities, identifying children with delays and providing valid information on early language ability (Fenson et al., 1993, 2000a, b, 2007; Wetherby and Prizant, 2002; Law and Roy, 2008; Wallace et al., 2015). Various parental report instruments, such as the Infant-Toddler Checklist (ITC) and different forms of Communicative Developmental Inventories (CDIs), are available for evaluating 2 year-old children’s communication and language skills (Wetherby and Prizant, 2002; Fenson et al., 2007). Still, to our knowledge, comparison studies on their usability are lacking. Thus, it is unclear, e.g., which measures provide the most representative information on emerging language capacity, including their predictive validity. Further, since different parental report instruments focus on different types of language and communication skills, such as vocabulary comprehension, gestures, production of sounds and words, and grammar, they may provide more comprehensive information when used together than separately. However, previous studies have not focused on this issue. In addition, most parental report instruments used to measure early language ability focus on expressive language, and the role of receptive language remains unclear. In all, further comparison information is needed on the usability of parental report instruments that measure the early language development of children at the end of the second year of life. The main aim of the present study was to investigate and compare the usability of the following three parental report instruments in a longitudinal setting: the *Infant-Toddler Checklist* of the Communication and Symbolic Behavior Scales Developmental Profile (ITC) and the *Short Form* and *Long Form* versions of the Finnish Communicative Development Inventories (FinCDI-SF, FinCDI-LF).

The Infant-Toddler Checklist (ITC) is a brief parental report instrument that can be used to screen prelinguistic and early language skills from 6 to 24 months (Wetherby and Prizant, 2002). It is a part of broader early social communication assessments called the Communication and Symbolic Behavior Scales Developmental Profile (Wetherby and Prizant, 2002). The ITC can be used to measure three developmental areas: social communication, expressive speech, and symbolic. These three composites include seven language predictors: emotion and eye gaze, communication, and gestures (social); sounds and words (speech); understanding and object use (symbolic). By evaluating these early language predictors together, it is expected to get valid information for early identification of delayed language skills even before spoken language becomes the primary communication method (Wetherby et al., 2002; Wetherby and Prizant, 2002; Watt et al., 2006; Eadie et al., 2010; Laakso et al., 2011; Wallace et al., 2015; Määttä et al., 2016; Stolt and Vehkavuori, 2018; Fäldt et al., 2021). The assessment of multiple prelinguistic skills may provide a broad overview of a child’s general language development both at the measurement point and over time (Wetherby et al., 2002; Wetherby and Prizant, 2002; Määttä, 2017; Borkhoff et al., 2022; Nurse et al., 2022). Results from earlier studies support the validity of ITC as a measure of prelinguistic and early language skills (Wetherby and Prizant, 2002; Wetherby et al., 2003). Later studies have also found that the ITC is a useful clinical tool for screening and predicting later language ability (Crais, 2011; Wallace et al., 2015; Määttä et al., 2016). Early language predictors measured using ITC between ages 1;0 and 2;0 have been found to be associated with receptive and expressive language outcomes at 2;0 and 3;0, in which the ITC explained 20–51% of the variance (Wetherby and Prizant, 2002; Wetherby et al., 2003). A previous study has also shown that measures of social communication between 18 to 21 months predict language outcomes at 2 and 3 years of age even better than expressive vocabulary production measures at 2;0 (Morgan et al., 2020). Regarding the Finnish language, comparable findings have been reported (Laakso et al., 2011; Määttä et al., 2016). The original American version has been translated and validated in Finnish with minor adaptations (Laakso et al., 2011). The norming study for the FinITC (*n* = 508 children) indicated significant, positive associations between the Speech and Symbolic composites when measured at 2;0 and language skills at 3;0 *r*-values ranged from 0.31 to 0.48; (Laakso et al., 2011). In addition, significant associations were found in later studies up to the age of 8, and the explanatory value was reported to range from 10.5 to 53.3% (Määttä et al., 2016).

The Communicative Development Inventories (CDIs) are parental report instruments that can be used to assess children’s language development between the ages of 8 and 30 months (Fenson et al., 2000b, 2007). Different forms of CDIs can be used to assess children’s early language development, including vocabulary comprehension, production, gestures, and grammar (for a review, see Law and Roy, 2008). A tool for slightly older children between 2;6 and 4 years is also available: the CDI III (Eriksson, 2017; Eriksson and Myrberg, 2023; Marchman et al., 2023; Stolt, 2023). The CDIs have been initially developed in American English. Adaptations have been made in almost 100 languages (The MacArthur-Bates Communicative Development Inventories, 2023). The original American Short Form version of the CDI (CDI-SF) includes three versions: an infant form for children between 8 and 18 months and two Toddler forms for children between 16 and 30 months (Fenson et al., 2000b). The measure has been adapted for various languages (e.g., for Spanish, Jackson-Maldonado et al., 2013; for Portuguese, Frota et al., 2016; for Swedish, Eriksson, 2017; for Finnish, Stolt and Vehkavuori, 2018; for Basque, Ezeizabarrena and Fernández, 2022), and it has been used for both clinical and research purposes (Fenson et al., 2000a; Dale et al., 2003; Pan et al., 2004; Can et al., 2013; Vehkavuori and Stolt, 2018, 2019; Lasorsa et al., 2021; Sansavini et al., 2021; Urm and Tulviste, 2021). Previous research has shown that the early expressive lexicon measured using the brief screening method, the short form version of CDI, is a valid predictor of later language skills. Early expressive lexical skills, when measured using the short-form versions of CDI, have been found to significantly explain variation in receptive language skills at 3;0 (Pan et al., 2004) and vocabulary, syntax, and semantics in children aged 5;6 to 6;8 (Can et al., 2013). The Short Form version of the CDI has also been adapted for the Finnish population (Stolt and Vehkavuori, 2018), the target population in this study. The FinCDI-SF has two different versions: an Infant Form for children aged 9–18 months and a Toddler Form for children aged 18–24 months. In previous studies, results for the FinCDI-SF Toddler version have been reported to be comparable to studies in other languages (Vehkavuori and Stolt, 2019; Vehkavuori et al., 2021). The results of the Toddler version were associated broadly with subsequent language skills, such as receptive and expressive language (Vehkavuori and Stolt, 2019) and lexical, phonological, morphological, and pre-literacy skills at 5;0 (Vehkavuori et al., 2021). Moreover, especially expressive lexicon at 1;6 and 2;0 explained 16–22% of the variation in general language ability at 5;0 (Vehkavuori et al., 2021).

The original American Long Form version of the Communicative Development Inventories (CDI-LF) can be used to collect information on different language domains, such as receptive and expressive vocabulary, gesture use, and syntactical skills (Fenson et al., 1993). The CDI-LF includes a Words and Gestures Form (WG, 8–16 months) and a Words and Sentences Form (WS, 16–30 months). The CDI-LF Words and Sentences Form used in the present study assesses early lexical ability, usage of words, emerging morphosyntactical skills, and sentence length. Different language versions of CDIs have been adapted to the language in question, and due to this, their content differs from each other. For example, the English form includes the complexity of the child’s multi-word utterances (Fenson et al., 2007), and the Finnish form includes the child’s usage of inflections (Lyytinen, 1999). The CDI-LF has been adapted for numerous languages (for a comparison study, see Bleses et al., 2008) and used widely (e.g., Fenson et al., 1993; Marchman and Martine-Sussmann, 2002; Stolt et al., 2009; Torppa et al., 2010; Eriksson et al., 2012; Simonsen et al., 2014; Marjanovič-Umek et al., 2017; Viana et al., 2017; Pérez-Pereira and Cruz, 2018; Cadime et al., 2019; Patrucco-Nanchen et al., 2019). As a result, there is plenty of evidence of the validity and usability of different languages in longitudinal studies (Fenson et al., 2007; Hurtado et al., 2014; Jago et al., 2023). For example, expressive vocabulary at 1;10 has been reported as a strong predictor of total vocabulary at 2;6 (Pérez-Pereira and Cruz, 2018), and early vocabulary is a good predictor of grammar acquisition, and it relates to the development of early communicative gestures (Marjanovič-Umek et al., 2017). Also, previous studies have reported associations between CDI-LF scores at 2;0 and different tests at 3;0 (Feldman et al., 2005; Korpilahti et al., 2016). Correlations between scores on vocabulary production and three longest utterances at 2;0 and scores on standardized tests at 3;0 have been reported to range from 0.32 to 0.39 (Feldman et al., 2005). The Long Form version of the CDI has been validated and normed for the Finnish population (Lyytinen, 1999) and used in longitudinal studies (e.g., Lyytinen et al., 2005; Stolt et al., 2014; Joensuu et al., 2021). One of these longitudinal studies reported that weak lexical skills at 2;6 indicate weak expressive language skills at 5;6 (Lyytinen et al., 2005). In addition, the risk for weak subsequent language skills increased if weak concurrent expressive language skills were accompanied by weak early receptive language skills (Lyytinen et al., 2005). Also, a previous longitudinal study showed that early weak language skills at 2;0 predicted later weak language skills at 5;0 in prematurely born children with very low birth weight (Stolt et al., 2014). Moreover, a recent follow-up study found significant associations between language skills at 2 years of corrected age and literacy skills at 7 years, also in preterm-born children (Joensuu et al., 2021). It was found that the small lexicon size assessed with the help of FinCDI-LF and the short mean length of the three longest utterances correlated significantly with literacy measures (*r*-values varied between 0.31 and 0.43).

Most 2 year-old children use words for communication, but variation is vast. At 2;0, children have acquired the basic lexicon of their native language, and the average lexicon size varies between 200 and 400 words (Fenson et al., 2007; Bleses et al., 2008; Stolt et al., 2008). Still, variation between individual children is extensive: some children have acquired only some words, whereas others have lexicons of over 600 words. Word combinations appear between 18 and 20 months, and grammatical development follows lexical development. Therefore, age 2;0 is a prominent age point for assessing especially lexical development. Roughly 90% of children use at least two-word combinations at 2;0, and some may use very long sentences (Fenson et al., 2000b, 2007; Stolt et al., 2009). Expressive vocabulary size is strongly associated with grammatical development (Conboy and Thal, 2006; Stolt et al., 2009). Further, weak language ability is often identified at 2;0 by assessing lexicon size with parental report instruments, such as CDIs (Feldman et al., 2005; Desmarais et al., 2008; Law and Roy, 2008). One criterion for weak language ability at this age is fewer than 50 expressive words in the lexicon or the lack of word combinations (Rescorla et al., 2005; Zubrick et al., 2007; Dollaghan, 2013; Hawa and Spanoudis, 2014; Farabolini et al., 2023). The other commonly used criterion is that the child’s expressive vocabulary size remains under the 10th percentile of the population in question (Girolametto et al., 2001; Heilmann et al., 2005; Desmarais et al., 2008; Rescorla and Dale, 2013). In the present study, both cut-off values are used.

The main aim of the present study is to compare and investigate the usability of three parental report instruments. The research questions were as follows: (1) Which one of the following parental report instruments when used at 2;0 has the strongest associations with receptive/expressive/total language ability when measured using the Reynell Developmental Language Scales (RDLS) at 3;6: Communication and Symbolic Behavior Scales, Developmental Profile, Infant-Toddler Checklist (ITC), the Short Form version of the Communicative Development Inventories (FinCDI-SF), the combined value of the ITC and FinCDI-SF or the Long Form version of the Communicative Development Inventories (FinCDI-LF)? (2) Does the receptive/expressive/general language ability at 3;6 differ between those children with weak vs. typical skills measured using the ITC/FinCDI-SF/FinCDI-LF at 2;0? (3) How does early receptive language, measured using RDLS at 2;0, contribute to the explanatory value of early parental report instruments: the ITC, FinCDI-SF, and FinCDI-LF?

## 2. Materials and methods

### 2.1. Participants

The participants were 68 (30 boys) typically developing, full-term (mean gestational week 40, SD 1.5), monolingual Finnish-speaking children. The families were invited to the study during a periodic health check-up at their local healthcare center at the age of 8 months. When the families were invited to the study, the participants had no diagnosis or suspicion of neurological disorders, such as hearing impairment, autism spectrum disorder, cerebral palsy, or cognitive delay. The parents were not known to have mental health issues or alcohol or drug abuse. All the parents had completed at least 9 years of compulsory schooling (Table 1).

**Table 1:** Education level of parents.

	Maternal, *n* (%)	Paternal, *n* (%)
Compulsory school	0 (0)	1 (1)
High school or vocational school	15 (22)	27 (40)
University of Applied Sciences degree	23 (33)	12 (18)
University degree	31 (45)	26 (39)

Information about one father’s education was missing. One of the participants had same-gender parents, whose education level information is under “maternal.”

This study is part of the Norming and Validation Study of Finnish Short Form Versions of the Communicative Development Inventories (FinCDI-SF, Sanaseula Study; principal investigator: the last author of the present article). The FinCDI-SF study has been approved by the Ethical Committee of the University of Turku. Each family signed written consent after being informed about the study. Parents received written information on their child’s language skills at both assessment points. Parents were instructed to contact their local child health clinic if a child had delayed language skills.

### 2.2. Measures at 2;0 and 3;6

The data were collected at two age points: 2;0 and 3;6. The following parental report instruments were used at 2;0: Communication and Symbolic Behavior Scales Developmental Profile, Infant-Toddler Checklist (ITC, Laakso et al., 2011; original version: Wetherby and Prizant, 2002), the Finnish Short Form version of Communicative Development Inventories (FinCDI-SF, Stolt and Vehkavuori, 2018; original version: Fenson et al., 2000a, b) and the Finnish Long Form version of the Communicative Development Inventories (FinCDI-LF, Words and Sentences form, Lyytinen, 1999; original version: Fenson et al., 1993, 2007). At 2;0 and 3;6, a formal test, the Reynell Developmental Language Scales III, was used (RDLS, Kortesmaa et al., 2001; original version: Edwards et al., 1997). All the measures have been normed and validated in the Finnish population.

The ITC consists of three composites: social communication (emotion and eye gaze, communication, gestures; 26 points), speech (sounds, words; 14 points), and symbolic (understanding, object use; 17 points) (Wetherby and Prizant, 2002; Laakso et al., 2011). The 24 items are rated on a 3–5-point scale. The maximum total score is 57 points. The cut-off value for the total score at 2;0 (i.e., weak skills) is ≤49 points, the lowest 10th percentile of the norming group for the measure. Typical development is defined as >49 points. The cut-off value for social communication is ≤21 points, for speech ≤12 points, and for symbolic ≤15 points (Laakso et al., 2011).

The FinCDI-SF Toddler questionnaire includes a wordlist of 100 words and one additional question (max 2 points: 0 = not yet, 1 = sometimes, 2 = often) about the child’s word combination usage (Stolt and Vehkavuori, 2018). Different lexical categories are represented in it, e.g., social-pragmatic words, nouns, and verbs. The relative share of the categories is parallel to that of the Finnish Long Form version. The FinCDI-SF is a briefer method compared to the Long Form CDI and is, therefore, more suitable for screening purposes. The cut-off value for weak expressive lexical skills at 2;0 is ≤12 words, the lowest 10th percentile of the norming group for the measure (Stolt and Vehkavuori, 2018).

The FinCDI-LF Words and Sentences form was used to gather information on early lexical ability, usage of words, emerging morphosyntactic skills, and sentence length at 2;0 (Lyytinen, 1999). The vocabulary score includes 595 items from different semantic categories. Different lexical categories are represented, e.g., social terms, nouns, and verbs. The usage of words is measured with five questions (max 5 points; 0 = does not use, 1 = uses). Morphosyntactic skills are measured based on the use of 16 different inflectional forms (max 32 points: 0 = not yet, 1 = sometimes, 2 = often). The ability to use word combinations is measured with one question (scored in the present study as max 1 point: 0 = does not use, 1 = uses sometimes or often). The mean length of the three longest utterances (M3L) calculated in morphemes is counted to get information on the utterance length of the children. The cut-off values at 2;0 were as follows: ≤30 words for expressive lexical skills, 2.06 for M3L, and 1.20 for inflectional forms (Lyytinen, 1999; Eklund, personal communication, 2017).^1^ These are the lowest 10th percentiles of the norming group for the measure; scores above these are defined as typical development skills.

The RDLS is a formal test (Edwards et al., 1997; Kortesmaa et al., 2001). In the present study, it was used to measure receptive language ability at 2;0 and receptive, expressive, and general language ability at 3;6. The adapted Finnish version has normative data from 1;10 to 7;0. The receptive scale measures the comprehension of lexical items (nouns, verbs, prepositions) and simple and complex sentences. The expressive scale measures the ability to use different vocabulary items and long sentences. The total score is 124 points: 62 points for receptive skills and 62 points for expressive skills. Raw points are converted into standard scores; mean 100 points, +/− 1 SD 15 points. Weak general language ability was defined as ≤85 standard scores, and typical language ability as >85 (Kortesmaa et al., 2001).

### 2.3. Data analysis

Pearson’s correlation coefficient values were used to test the associations between the results of early parental report instruments at 2;0 and general language ability at 3;6. Two sum scores were created to analyze the associations of the combined values of the measures: (1) ITC total score + FinCDI-SF vocabulary score and (2) vocabulary + M3L of the FinCDI-LF. The sum scores were created based on the *z*-values of each measure. The combined value for the ITC total score and the FinCDI SF was used to test whether it is possible to derive more comprehensive information on a child’s early language with the help of two different types of brief parental report instruments together than when using either instrument alone. The combined value for the vocabulary score and the M3L value of the FinCDI-LF was used to test what kind of information these two values provide together compared to when used alone. The standard scores for the RDLS were used in all statistical analyses.

The Mann–Whitney *U* test was used to compare the differences between two groups; children with weak language skills at 2;0 and children with typical language skills at 2;0. Children with weak skills were defined based on the normative values for each measure (≤10%), ≤50 first words, or word combinations not used.

Six linear regression models were created to investigate how much of the variance in general language ability at 3;6 can be explained by the results of the parental report instruments when used at 2;0. In addition, the role of early receptive language was also investigated when measured using the RDLS at 2;0 in these models. The total score of the RDLS measured at 3;6 was used as an outcome variable in all models. The first model used the ITC and FinCDI-SF total scores as predictors. In the second model, the receptive language ability measured at 2;0 was added as a predictor. The FinCDI-LF vocabulary score was used as a predictor in the third model, and the receptive language score was included in the fourth model. In the fifth model, the M3L value of the FinCDI-LF was used as a predictor, and in the sixth model, receptive language ability was included. The following background factors were included in all models: gender and maternal education (four groups, Table 1). All analyses were carried out using IBM SPSS Statistics for Macintosh, version 28.0.

## 3. Results

### 3.1. Data description

The descriptive statistics for the ITC, FinCDI-SF, FinCDI-LF, and RDLS are presented in Table 2. The mean value of the ITC was 52.5 (SD 3.2), and most children had typical skills when measured using ITC. Eleven children (16%, 8 boys) had a total score at or below the cut-off value. Fifteen children (22%, 8 boys) had weak social communication skills, seven children (10%, 5 boys) had weak speech skills, and four children (6%, 4 boys) had weak symbolic skills. When the FinCDI-SF values were considered, a considerable variation in expressive lexical development was detected. Children used roughly half of the items included in the measure. Six children (9%, 4 boys) had weak lexical skills.

**Table 2:** Descriptive statistics for test results measuring language skills at 2;0 (*n* = 68).

Method		Mean (SD)	Median	Min.–Max.
ITC				
	Social communication	22.7 (2.6)	23.0	17–26
	Speech	13.2 (1.2)	14.0	8–14
	Symbolic	16.6 (0.6)	17.0	15–17
	Total score	52.5 (3.2)	53.0	44–57
FinCDI-SF				
	Expressive vocabulary	57.1 (26.7)	60.5	4–100
FinCDI-LF				
	Expressive vocabulary	271.3 (148.3)	294.0	10–528
	Word use	8.4 (1.8)	9.0	1–10
	Inflectional forms	16.3 (9.7)	9.7	0–32
	M3L^*^	6.6 (3.3)	7.0	1–17
RDLS				
	Receptive	107.7 (16.2)	107.0	73–142

ITC, Infant-Toddler Checklist of the Communication and Symbolic Behavior Scales Developmental Profile; FinCDI-SF, Finnish Short Form versions of the Communicative Development Inventories; FinCDI-LF, Finnish Long Form versions of the Communicative Development Inventories; RDLS, Reynell Developmental Language Scales III (standard scores). ^*^One missing value.

Also, based on the FinCDI-LF, the children’s expressive lexical skills varied considerably (Table 2). The mean value of lexicon size for the FinCDI-LF was 271 words (SD 148.3). Four children (6%, 4 boys) had weak lexical skills when the 10th percentile value of the norming sample for the FinCDI-LF was used as a cut-off value. In addition, 7 children (10%, 4 boys) had <50 words in their lexicons at 2;0. Children used roughly nine morphological inflections. Three children (4%, 2 boys) had weak skills in using inflectional forms. The mean length of the three longest utterances was 7, and seven children (10%, 5 boys) used very short utterances based on the M3L. Most children (91%, 25 boys) had started to use word combinations. Six children (9%, 5 boys) did not use word combinations at 2;0.

In the RDLS, most children (>85 standard scores, *n* = 60, 88%, 25 boys) had typical receptive language ability at 2;0 (Table 2). However, there was considerable variation in receptive language ability at 2;0. Eight children (12%, 5 boys) had weak receptive language skills.

The descriptive statistics for language skills at 3;6 are presented in Table 3. The mean and median values were within normal variation. There was significant variation in the language abilities of the participants at 3;6. There were five children (7%, 5 boys) with weak language skills. Of these five children, three had weak receptive skills, three children had weak expressive skills, and three children had weak general language ability.

**Table 3:** Descriptive statistics for test results (standard scores) measuring receptive, expressive, and general language skills at 3;6 using the RDLS (*n* = 68).

Method	Mean (SD)	Median	Min.–Max.
RDLS			
Receptive	107.4 (12.6)	109.0	50–134
Expressive	103.5 (12.8)	104.0	75–131
Total score	105.4 (13.0)	107.0	55–130

RDLS, Reynell Developmental Language Scales III (standard scores).

### 3.2. Association between early communication and language skills and language ability at 3;6

Most correlations between the results of early parental report instruments and later language ability were statistically significant (Table 4). The total score of the ITC, when used at 2;0, correlated positively and significantly with receptive, expressive, and general language ability at 3;6 (*r*-values varied between 0.26 and 0.34, and *p*-values varied between 0.01 and 0.04). From the separate composites of the ITC, only the social communication composite was significantly associated with later receptive language. However, speech and symbolic composites were associated with later expressive language ability (Table 4). The strongest correlations were found between the speech composite and later expressive language ability. Regarding the FinCDI-SF, expressive vocabulary at 2;0 correlated clearly and relatively evenly with later receptive and expressive language ability at 3;6 (*r*-values varied between 0.32 and 0.38, and *p*-values varied between 0.00 and 0.01).

**Table 4:** Correlations (Pearson’s *r*-values and *p*-values) between early communication and language skills at 2;0 and language skills at 3;6 (*n* = 68).

Method	RDLS standard scores at 3;6						Receptive		Expressive		Total score		*r*	*p*	*r*	*p*	*r*	*p*
ITC						
Social communication	**0.28**	**0.02**	0.09	0.49	0.23	0.06
Speech	0.20	0.10	**0.36**	**0.00**	**0.31**	**0.01**
Symbolic	0.10	0.41	**0.27**	**0.03**	0.20	0.10
Total score	**0.32**	**0.01**	**0.26**	**0.04**	**0.34**	**0.01**
FinCDI-SF						
Expressive vocabulary	**0.32**	**0.01**	**0.34**	**0.00**	**0.38**	**0.00**
Sum score of ITC + FinCDI-SF	**0.38**	**0.00**	**0.35**	**0.00**	**0.42**	**0.00**
FinCDI-LF						
Expressive vocabulary	**0.38**	**0.00**	**0.44**	**0.00**	**0.46**	**0.00**
Word use	0.15	0.22	**0.27**	**0.03**	0.22	0.07
Inflections	**0.24**	**0.05**	**0.29**	**0.02**	**0.29**	**0.02**
M3L	**0.32**	**0.01**	**0.46**	**0.00**	**0.42**	**0.00**
Sum score of LF vocabulary + LF M3L	**0.37**	**0.00**	**0.49**	**0.00**	**0.47**	**0.00**
RDLS						
Receptive	**0.52**	**0.00**	**0.62**	**0.00**	**0.68**	**0.00**

Significant correlations (*p* < 0.05) are marked in bold. ITC, Infant-Toddler Checklist of the Communication and Symbolic Behavior Scales Developmental Profile; FinCDI-SF, Finnish Short Form versions of the Communicative Development Inventories; FinCDI-LF, Finnish Long Form versions of the Communicative Development Inventories; RDLS, Reynell Developmental Language Scales III.

When the variables of FinCDI-LF were considered, expressive vocabulary, word use, inflectional forms, and the mean length of utterances were associated significantly and positively with later general language ability (Table 4). Statistically significant positive correlations were found between the FinCDI-LF variables and later receptive, and particularly expressive, language ability (*r*-values varied between 0.24 and 0.46, and *p*-values varied between 0.00 and 0.05). The strongest association with general language ability at 3;6 was found between expressive vocabulary and M3L when measured using the FinCDI-LF (*r*-values varied between 0.29 and 0.47, and *p*-values varied between 0.00 and 0.02).

The combined value of the ITC and FinCDI-SF correlated more strongly with later language scores than the result of each of the measures separately (Table 4). The strongest correlation coefficient values were found between the receptive language ability, measured using the RDLS at 2;0, and receptive, expressive, and general language ability, measured using the RDLS at 3;6 (*r*-values varied between 0.52 and 0.68, and *p*-values were 0.00).

### 3.3. Comparison of language ability at 3;6 in children with weak vs. typical skills at 2;0

Comparisons between the weak- vs. typical-skills groups, when defined using different parental report instruments at 2;0 and regarding children’s language ability at 3;6, are presented in Table 5. Based on the comparisons, many significant differences were found. Most of the investigated variables showed significant differences between the two groups (weak vs. typical skills at 2;0) in expressive and general language ability when measured using the RDLS at 3;6. In other words, those children with weak skills at 2;0 still had weaker skills than the rest of the group at 3;6.

**Table 5:** Receptive, expressive, and general language ability at 3;6 in children with weak vs. typical skills measured using the ITC, FinCDI-SF, and FinCDI-LF at 2;0 (Mann–Whitney U test).

	RDLS standard scores at 3;6				Mean (SD)	Mean (SD)	*U*	*p*	Weak skills group at 2;0	Typical skills group at 2;0		
	ITC	ITC		
Receptive	98.9 (18.8)	109.0 (10.5)	197.50	0.053
Expressive	95.3 (13.9)	105.1 (12.1)	189.50	**0.039**
Total score	94.6 (17.4)	107.5 (11.0)	161.00	**0.011**
	FinCDI-SF	FinCDI-SF		
Receptive	98.5(9.2)	108.2 (12.6)	81.00	**0.021**
Expressive	89.8 (13.1)	104.9 (12.1)	73.00	**0.012**
Total score	92.8 (9.7)	106.6 (12.7)	57.00	**0.003**
	FinCDI-LF (vocabulary)	FinCDI-LF (vocabulary)		
Receptive	99.8 (11.6)	107.9 (12.6)	95.00	0.178
Expressive	84.3 (9.8)	104.8 (12.1)	22.50	**0.002**
Total score	89.8 (9.8)	106.4 (12.6)	29.00	**0.006**
	≤50 first words	>50 first words		
Receptive	100.3 (9.7)	108.2 (12.7)	116.50	0.050
Expressive	91.6 (12.8)	104.9 (12.2)	99.00	**0.021**
Total score	94.9 (10.4)	106.7 (12.8)	83.50	**0.009**
	No word combinations	Word combinations		
Receptive	99.8 (9.4)	108.1 (12.7)	95.00	**0.048**
Expressive	92.7 (15.1)	104.6 (12.2)	105.00	0.079
Total score	95.2 (11.3)	106.4 (12.8)	82.00	**0.024**
	FinCDI-LF (M3L)	FinCDI-LF (M3L)		
Receptive	97.4 (8.4)	108.6 (12.7)	71.50	**0.004**
Expressive	91.4 (13.8)	105.2 (12.0)	91.00	**0.015**
Total score	93.3 (10.3)	107.1 (12.6)	66.50	**0.003**

Weak skills were defined as the ≤10th percentile of the measure in question. Standard scores of the RDLS at 3;6 are presented. Group comparisons are also presented. Significant correlations are marked in bold. ITC, Infant-Toddler Checklist of the Communication and Symbolic Behavior Scales Developmental Profile; FinCDI-SF, Finnish Short Form versions of the Communicative Development Inventories; FinCDI-LF, Finnish Long Form versions of the Communicative Development Inventories; RDLS, Reynell Developmental Language Scales III.

### 3.4. Explanatory value of early parental report instruments and the role of receptive language ability

The regression models used to investigate the explanatory value of the results of parental report instruments are presented in Table 6. All models were statistically significant (*p* < 0.00). Regarding the models that did not include receptive language ability at 2;0 as a predictor, the explanatory values varied between 18 and 22%. When receptive language ability was added to the models as a predictor, the explanatory values of the models increased considerably (46–48%). Background factors were not statistically significant in any of the models. The best model for explaining the general language ability at 3;6 included three variables: the ITC, the FinCDI-SF, and the receptive score of the RDLS at 2;0. This model explained 48% of the variation in general language ability at 3;6.

**Table 6:** The explanatory value of early parental report instruments and receptive language ability measured at 2;0 regarding general language ability at 3;6 – the regression models.

Model information	Predictors at 2;0	Beta	*t*	Sig.
Model 1				
Outcome variable: RDLS total	ITC	0.20	1.56	0.12
*F*(4,63) = 4.57, *p* < 0.00	FinCDI-SF	0.25	1.96	**0.05**
*R*^2^_adj._ = 0.18	Gender	−0.18	−1.56	0.12
	Maternal education	0.10	0.89	0.38
Model 2				
Outcome variable: RDLS total	ITC	0.20	2.06	**0.04**
*F*(5,62) = 13.49, *p* < 0.00	FinCDI-SF	−0.04	−0.38	0.70
*R*^2^_adj._ = 0.48	RDLS receptive	0.64	6.19	**0.00**
	Gender	−0.12	−1.33	0.19
	Maternal education	−0.02	−0.22	0.82
Model 3				
Outcome variable: RDLS total	FinCDI-LF (vocabulary)	0.41	3.64	**0.00**
*F*(3,64) = 7.11, *p* < 0.00	Gender	−0.18	−1.63	0.11
*R*^2^_adj._ = 0.22	Maternal education	0.07	0.07	0.54
Model 4				
Outcome variable: RDLS total	FinCDI-LF (vocabulary)	0.12	1.12	0.27
*F*(4,63) = 15.56, *p* < 0.00	RDLS receptive	0.60	5.56	**0.00**
*R*^2^_adj._ = 0.47	Gender	−0.14	−1.50	0.14
	Maternal education	−0.04	−0.39	0.70
Model 5				
Outcome variable: RDLS total	FinCDI-LF (M3L)	0.38	3.21	**0.00**
*F*(3,63) = 5.78, *p* < 0.00	Gender	−0.21	−1.82	0.07
*R*^2^_adj._ = 0.18	Maternal education	−0.01	−0.05	0.96
Model 6				
Outcome variable: RDLS total	FinCDI-LF (M3L)	0.10	0.95	0.35
*F*(4,62) = 15.11, *p* < 0.00	RDLS receptive	0.62	5.83	**0.00**
*R*^2^_adj._ = 0.46	Gender	−0.14	−1.50	0.14
	Maternal education	−0.07	−0.73	0.47

ITC, Infant-Toddler Checklist of the Communication and Symbolic Behavior Scales Developmental Profile; FinCDI-SF, Finnish Short Form versions of the Communicative Development Inventories; FinCDI-LF, Finnish Long Form versions of the Communicative Development Inventories; RDLS, Reynell Developmental Language Scales III.

## 4. Discussion

The present study investigated and compared the associations of three parental report instruments when used at 2;0 and language skills at 3;6. In addition, it was also investigated if receptive/expressive/general language ability at 3;6 of those children with weak skills at 2;0 differed from the language ability of those children with typical language skills at 2;0. Further, the role of receptive language ability when assessing the possible predictive value of early language ability using the three parental report instruments was also analyzed. Most of the correlations between the results of early measures and later language ability were positive and statistically significant. The correlation coefficient value between the combined value of the ITC and FinCDI-SF and a later language score was higher than the one for individual measures. Early receptive language skills correlated clearly and significantly with later receptive, expressive, and general language abilities. In general, the participants with weak language skills at 2;0, such as vocabulary, word combinations, or mean length of the three longest utterances, had weaker language skills at 3;6 compared with the participants with typical early language development. All regression models, which were modified to investigate the possible explanatory value of parental report instruments when used at 2;0, significantly explained the variability in language skills at 3;6. However, the best models included the receptive language score at 2;0 as an explaining factor.

The parental report instruments are widely used, and the findings of this study support their being valuable tools for assessing early language skills and predicting later language ability. The results of all three parental report instruments used in this study were significantly associated with later language ability. These findings align with various studies and strengthen previous results (Feldman et al., 2000; Wetherby et al., 2002; Pan et al., 2004; Can et al., 2013; Wallace et al., 2015; Vehkavuori and Stolt, 2019; Vehkavuori, 2021). For example, the ITC is an acceptably sensitive and specific screening instrument for parents to complete and for early identification of language delays or disorders (Wetherby et al., 2003; Wallace et al., 2015). Further, in this study, the different composites of ITC provided slightly different information on later language ability. This finding supports the results of previous studies, which showed that the social communication composite correlated only with receptive language skills at 3;6, and speech and symbolic composites were associated significantly with expressive language skills at 3;6 (Laakso et al., 2011; Määttä et al., 2016). In addition, in this study, the FinCDI-SF correlated equally well with receptive and expressive language skills at 3;6. This finding confirms that the FinCDI-SF can be used to predict later receptive and expressive skills. It is in line with a previous study which has shown associations between early lexicon and general language ability at 5;0 (Vehkavuori et al., 2021).

Regarding the FinCDI-LF results, the strong correlations between expressive vocabulary and mean length of the three longest utterances with later language skills at 3;6 indicate that these measures are reliable indicators of language development. This finding is in line with previous research that has shown the importance of early expressive lexicon in predicting later lexical skills (Pan et al., 2004; Can et al., 2013; Pérez-Pereira and Cruz, 2018). Moreover, the present study found that the strongest correlation between early language measures and later language ability was observed when the combined value of expressive vocabulary size and M3L was used. This finding suggests that early language assessments should consider both expressive vocabulary development and morphosyntactic skills. In other words, it is important to evaluate a child’s ability to use morphosyntax when assessing their language ability at 2;0.

To our knowledge, longitudinal information on the combined value of the ITC and the short form version of the CDI, the FinCDI-SF in this case, has not been previously presented (see however Vehkavuori, 2021). Thus, our finding that the combined value of the ITC and the short form version of the CDI provides more comprehensive information on the language ability in young children than when these instruments are used separately, is novel. This combination was used to derive as comprehensive information on a child’s early language skills as possible with the help of two different types of brief parental report instruments. Our finding suggested that more representative information on early language development could be derived when two short instruments were used together than if used separately. This finding may be explained by the fact that these two instruments have been modified differently and they assess different domains of language, communication, and symbolic skills. Therefore, more comprehensive information on early language development can be derived when two different instruments are used together than if used separately.

Significant differences in language ability at 3;6 were found based on the comparison between children with weak vs. typical skills defined using the parental report instruments at 2;0. This result is parallel with studies examining late talkers or children who exhibit delayed expressive language skills, which have shown that weak early language skills are a risk factor for later weak language skills (Rescorla, 2011). However, most late-talking children catch up with their peers by age three, and a subset of them continue to struggle with language development. More longitudinal studies are needed to evidence outcomes of weak early expressive skills and to identify precursors of persistent language difficulties (Rescorla, 2011; Rescorla and Dale, 2013). The present study’s finding about differences between the two groups is consistent with a previous study which showed that children who produced word combinations at 2;0 had better expressive language skills at age 8 (Poll and Miller, 2013). However, when interpreting the findings of the present study, it is important to take into consideration that the mean values of the weak group defined at 2;0 were within the typical variation at 3;6. This could be due to the fact that the present sample included only typically developing and generally healthy children without any known specific diagnoses which could have impacted the language ability of the weak group. Suppose the sample included children with language difficulties or more children with weak skills; in that case, one may assume that even more evident differences could have been detected between the groups at 3;6. The uneven number of participants in weak vs. typically developing groups may also have influenced the finding. Still, despite these factors, our result supports the view that the parental report instruments which were used in the present study could identify those with weaker language skills from those with better skills.

All regression models that included results from parental report instruments as predictors explained later language ability statistically significantly, which is consistent with previous studies (Law and Roy, 2008; Wallace et al., 2015). However, when receptive language ability measured using the RDLS at 2;0 was included in the model as a predictor, the explanatory value of the model increased considerably. This finding emphasizes the importance of early receptive language ability when assessing the language skills of 2 year-old children. Previous research has demonstrated that early receptive language is a significant predictor of later expressive language skills (Fisher, 2017), while early expressive vocabulary alone may not be the most reliable indicator of persistent language difficulties (Dollaghan, 2013). To our knowledge, the combined explanatory value of the parental report instruments, which primarily focus on expressive language ability, and receptive language measured using formal tests has not been previously investigated. Thus, our novel result provides important information to the field.

Regarding the strengths of the present study, one may conclude that this study provides novel comparison information on parental report instruments. While the difference between using two brief parental report instruments in parallel vs. separately was modest, even further information can be derived by using them together. Both receptive and expressive language ability at two age points was taken into consideration, which is another strength of this study. The ITC and the CDIs measure different domains of language, communication, and symbolic skills, which allows clinicians and researchers to get more comprehensive information on early language skills when used together. A limitation of this study is the small group of children with weak language skills, which may have affected the comparisons of the two subgroups. A larger group of children with weak language skills at 2;0 would have allowed investigation of the differences between the groups at 3;6 in a more detailed manner.

The present longitudinal study aimed to investigate early language development in children in a longitudinal setting, with a focus on comparing different parental report instruments. The study’s clinical implications are twofold. First, our findings suggest that clinicians should consider using multiple parental report instruments in parallel to obtain more representative information about early language development. Second, it is important to assess early receptive language skills at 2;0 as this study indicates. However, many existing parental report instruments primarily focus on expressive language. This study underscores the value of validated parental report instruments while also highlighting the need for new instruments that capture receptive language development more accurately.

In conclusion, the present study contributes novel insights into the comparative usability of three different parental report instruments. Our results demonstrate that utilizing two brief parental report instruments in parallel yields more comprehensive information on later language skills than using either instrument alone. Additionally, significant differences in later language skills were observed between children with weak vs. typical skills at 2;0, as defined by the three different parental report instruments. Our findings suggest that parental report instruments can provide a useful indication of later language skills, at least to some extent. Furthermore, our study highlights the significance of assessing receptive language skills in 2 year-old children.

## Data availability statement

The datasets presented in this article are not readily available because of ethical and privacy issues. Requests to access the datasets should be directed to SSt, suvi.stolt@helsinki.fi.

## Ethics statement

The studies involving human participants were reviewed and approved by the Ethics Committee of the University of Turku. Written informed consent to participate in this study was provided by the participants’ legal guardian/next of kin.

## Author contributions

SSu, S-MV, KS-H, and SSt contributed to the conception and design of the study. SSu, S-MV, and SSt organized the database. SSu performed the statistical analyses with the help of a statistician, wrote the first draft of the manuscript, and finalized the manuscript. S-MV, KS-H, and SSt critically reviewed the manuscript for important intellectual content. All authors contributed to the article and approved the submitted version.

## Funding

This study is supported by the Finnish Concordia Fund. The Sanaseula Study has been supported by the Päivikki and Sakari Sohlberg Foundation and the Jenny and Antti Wihuri Foundation. The publication fee is supported by the University of Helsinki.

## Conflict of interest

The authors declare that the research was conducted in the absence of any commercial or financial relationships that could be construed as a potential conflict of interest.

## Editor’s note

Maria-José Ezeizabarrena edited the article in collaboration with Melita Kovacevic, University of Zagreb, Zagreb, Croatia.

## Publisher’s note

All claims expressed in this article are solely those of the authors and do not necessarily represent those of their affiliated organizations, or those of the publisher, the editors and the reviewers. Any product that may be evaluated in this article, or claim that may be made by its manufacturer, is not guaranteed or endorsed by the publisher.

## Footnotes

^1^Eklund, K. (2017). Email to Suvi Stolt, 6 February (personal communication).
